# Impact of handwriting training on fluency, spelling and text quality among third graders

**DOI:** 10.1007/s11145-018-9825-x

**Published:** 2018-01-29

**Authors:** Sibylle Hurschler Lichtsteiner, Werner Wicki, Péter Falmann

**Affiliations:** University of Teacher Education, Töpferstrasse 10, 6004 Lucerne, Switzerland

**Keywords:** Handwriting, Fluency, Automaticity, Text quality, Handwriting training

## Abstract

As recent studies and theoretical assumptions suggest that the quality of texts composed by children and adolescents is affected by their transcription skills, this experimental field trial aims at investigating the impact of combined handwriting/spelling training on fluency, spelling and text quality among normally developing 3rd graders (N = 175). In addition to the combined handwriting/spelling training group, the sample includes two other intervention groups, a handwriting training group and a spelling training group as well as a reading fluency training control group. The participating teachers (N = 11) and their students were randomly assigned to the different intervention and control conditions, which were scheduled to last 20 units (each unit lasts 15 min) distributed over 5 weeks (4 units/week). Data collection was administered both before (pre-test) and after (post-test) the intervention as well as 3 months later (follow-up). Measures included group-administered tests and tasks (spelling, visuo-motor integration, copy task, and composing) and individually administered tests and tasks (working memory and several handwriting tasks on the digitizing tablet). As handwriting automaticity (measured on the digitizing tablet) was already high at the beginning of the study, the intervention was not able to improve it further. In consequence, an intervention effect on the quality of composed texts was not observed. Instead, text quality was predicted by working memory, fluency, spelling, and gender irrespective of the intervention.

## Introduction

Handwriting is a cultural skill and is taught and used in every school around the world, as well as applied in leisure activities and labour contexts later on despite the fact that keyboard typing is recently becoming more and more prevalent early in life.

### Models of handwriting

Most scholars understand handwriting as a complex neuromotor skill involving cognitive and motor processes sequentially as well as simultaneously. Contemporary models assume that these processes are controlled by a hierarchic architecture of both central and peripheral components (Kandel, Peereman, & Ghimenton, 2013) operating partially in a parallel mode (e.g. Kandel, Peereman, Grosjacques, & Fayol, 2011; Van Galen, 1991; Van Galen, Meulenbroek, & Hylkema, 1986).

According to Hayes (2012a, b), composing (handwriting as well as typing) consists of higher and lower level processes whereby handwriting is conceptualized within the low-level transcription process that follows planning and translating. While higher-level processes include ideation, choice of words (semantic coding) and syntax, lower-level processes refer to the retrieval of orthographic and allographic representations as well as the activation of neuromuscular networks and the subsequent execution of fine-motor movements. In general, higher order processes precede lower order processes, however, at least some processes are supposed to be synchronic, i.e. a writer executes an allograph while planning to write the next syllable during continuous handwriting (Kandel, Álvarez, & Vallée, 2006; Kandel et al., 2011). In addition, recent research indicates that some central processes are still ongoing during movement execution, i.e. showing that the writing system functions in a cascading manner (Delattre, Bonin, & Barry, 2006; Kandel et al., 2013; Olive, Alves, & Castro, 2009; Olive & Kellogg, 2002; Roux, McKeff, Grosjacques, Afonso, & Kandel, 2013). A writer can synchronously be engaged in transcribing a word and processing the next word. Therefore, interferences between different levels of handwriting are likely to occur during movement execution (Fayol & Lété, 2012).

### Automaticity of handwriting, working memory, and text quality

Low level components of handwriting implicate an integration and coordination of spelling knowledge, allographic representations and the execution of fine-motor movements (Berninger, 2009; Berninger & Swanson, 1994). For young children at the very beginning of their handwriting experience, each of these tasks is attention demanding and working memory resource consuming (Graham, Berninger, Abbott, Abbott, & Whitaker, 1997). The more this integration is automated, the lower the amount of the cognitive load is generally expected to be necessary (Bourdin & Fayol, 1994; Fayol & Lété, 2012) since handwriting automation is assumed to save resources for the remaining processes involved in text production (Grabowski, 2010; Graham et al., 1997; McCutchen, 1996, 2011). Children whose handwriting is not yet automated are easily overwhelmed with the more complex tasks of text production because of the demands on cognitive resources imposed on them, resulting in poor text production outcomes (e.g. Berninger & Swanson, 1994; Christensen & Jones, 1999; Graham, 1990). Accordingly, a training of handwriting over 8 weeks evidenced improvements both of the quality and quantity of written texts among adolescents suffering from poor orthographic-motor integration while the matched control group (whose students were instructed to write a diary) did not improve noticeably (Christensen, 2005). As Kim and Schatschneider (2016) demonstrated that working memory is a strong predictor of text quality already among first graders, handwriting training seems to be a promising method to improve the text quality early in the school career. Over the last years, this assumption could be confirmed for children with and without disabilities: automaticity trainings of transcription skills as handwriting lead to improvements in both text length and quality (Graham, McKeown, Kiuhara, & Harris, 2012). In their intervention study among Portuguese second graders, Alves et al. (2015) showed that a handwriting intervention group reached not only better handwriting fluency and text quality, but performed longer bursts with shorter pauses while writing texts when compared to keyboarding students. The handwriting training lasted over 10 weeks and included a pure functional graphomotor “warm up”, followed by more and more challenging exercises, i.e. using the trained letters within words, sentences and stories. The comparison groups received similar trainings in spelling or keyboarding.

### Working memory, automaticity of handwriting and spelling

There is also some evidence that cognitive load is not only devoted to the higher-level processes of writing, but also to transcription such as spelling. Spelling requires the retrieval of orthographic knowledge about the correct letters and their sequences in spoken or written words involving both orthographic long-term memory and working memory resources (Buchwald & Rapp, 2009). According to Tainturier and Rapp (2001), two sets of processes are involved in spelling that do not function independently from one another: among frequent words the letter sequences are directly retrieved from the so-called orthographic lexicon (lexical route), whereas for rare or novel words, the letter sequences are derived by a phonology to orthography conversion system (sublexical route). From this point of view, we assume that spelling capability is affected by the phonological component of working memory. However, it has been demonstrated that the visual-spatial modality of working memory has an additional effect on children’s spelling (Bourke, Davies, Sumner, & Green, 2014; Fischbach, Könen, Rietz, & Hasselhorn, 2014). Sumner, Connelly, and Barnett (2013, 2014) have investigated the impact of spelling capabilities on handwriting production in a copy task by comparing children with and without dyslexia. They found many more within-word pauses (and constrained productivity and fluency for that reason) among the dyslectic children compared to the spelling-ability matched children. Interestingly, children with dyslexia were able to write fast, but paused longer than the controls within-words, probably due to cognitive demands required by spelling information processing and poor lexical representations. It can therefore be concluded that handwriting fluency, as well as working memory, are both positively related to spelling capacity.

### Handwriting and visual-motor integration

Visual-motor integration (VMI) skills proved to be associated with legibility (e.g. Tseng & Murray, 1994; Volman, van Schendel, & Jongmans, 2006), however, research using the Developmental Test of VMI did not find an association with handwriting difficulties in young children (Marr & Cermak, 2002) or with handwriting dysfunction in older children (Goyen & Duff, 2005). Nevertheless, there is some evidence that VMI is related to academic performance in reading and writing (Kulp, 1999; Sortor & Kulp, 2003). Therefore, we included this variable to control for its impact on text quality.

### Gender effects

Handwriting fluency, automaticity and working memory are not the only factors influencing the quality of texts produced by children. Research has revealed that girls produce better and longer texts than boys (e.g. Kim, Al Otaiba, Wanzek, & Gatlin, 2015), therefore, the gender gap is an additional factor that has to be considered when predicting text quality. Berninger and Fuller (1992) investigated first, second and third graders and found that girls produced more words and clauses in narrative and expository composition than boys. Therefore, gender differences in transcription skills could at least partly explain the text quality differences. In addition, regarding the fact that girls have more positive attitudes than boys towards writing (Knudson, 1995; Lee, 2013), such motivational factors have explained additional variance in text quality in some studies (e.g. Knudson, 1995) but not in others (e.g. Graham, Berninger, & Fan, 2007).

### Training of transcription skills

Because of its important role as a transcription skill, handwriting has to be trained not only to become as legible as possible, but also to become more and more fluent. Fluency of handwriting means that a person is able to write in an automated way so that he or she does not have to think about how to form a letter or how to join movements of frequently used syllables. This results in an economic and fast way of personal handwriting. Automated movements show a particular shape of a smooth and regular course of velocity control: the ideal handwriter needs only one increase and decrease of speed per stroke unit (Mai & Marquardt, 1999).

As shown by Graham, Berninger, Weintraub and Schafer (1998) and by Wicki, Hurschler Lichtsteiner, Saxer Geiger and Müller (2014), the development of both legibility and fluency of handwriting is not yet finished at the end of the second grade. There is more training needed over the following years of schooling. In their meta-analysis, Troia and Graham (2009) published 17 recommendations about the most efficient ways of teaching handwriting, e.g. that in the primary grades, 75–100 min per week should be used for handwriting instruction, that letters sharing common strokes (e.g., a, d, and g) should be grouped together, and that the children should be monitored and immediately get help to form letters better when they are illegible. Teachers are requested to allow children to develop their own handwriting style and to ask them to self-evaluate their handwriting and to improve it continuously.

While traditional handwriting lessons used to focus on legibility of handwriting, the importance and the knowhow of teaching handwriting fluency is not yet well established. Santangelo and Graham (2016) recently found that in general, handwriting training is more efficient than no treatment. Individualizing instruction and using digital technology improved legibility, but no effects were found for fluency. A study by Berninger and co-authors (Berninger et al., 2006) on different ways to teach letters is important to understand why it is not useful to let children just copy letters directly from a model: In order to gain an automatized motor program, children need to trace a letter by visual arrows, getting verbal mediation, but afterwards, they are supposed to reproduce it from memory. Troia and Graham (2009) recommend further trainings in fluency by frequent writing and speed trials. Isolated motor trainings are inferior to handwriting training (Santangelo & Graham, 2016), accordingly, insights from promoting motor learning, e.g. the model of differential motor learning by Frank, Michelbrink, Beckmann and Schöllhorn (2008) or the principles of Neuromotor Task Training (Schoemaker & Smits-Engelsman, 2005) have been included in handwriting education (Jurt Betschart & Hurschler Lichtsteiner, 2017) and therapy. However, it has not yet been examined if they are really enhancing handwriting fluency among children with motor disabilities. A meta-analysis by Hoy, Egan, and Feder, (2011) proved that instructions of at least 20 lessons were effective, but the interventions focused on treatments by occupational therapist for children with handwriting difficulties.

So far, there are only a few German studies investigating children’s handwriting (e.g. Grabowski, Weinzierl, & Schmitt, 2010; Mahrhofer, 2004; Nottbusch, 2008; Sattler & Marquardt, 2010; Speck-Hamdan, Falmann, Hess, Odersky, & Rüb, 2016) and almost none looking at the link between transcriptions skills and text quality. This is unsatisfying because on the one hand German language has a more complex syllabic structure than Romance languages, as depicted by Seymour, Aro, and Erskine (2003), but on the other hand the orthographic depth of the German language is shallower than that of English for instance, so findings of previous studies in other languages may not be directly transferrable.

In regard to written language acquisition in German-speaking countries, there are different ways of learning how to read and write in general and how to write by hand in particular.

In the German-speaking part of Switzerland, a new handwriting style has been taught since 2001 in some schools locate. After positive research results confirming that children with the new handwriting type were able to write more legibly and fluently and showed more motivation to write than with the former one (Wicki & Hurschler Lichtsteiner, 2014), the so-called “Basisschrift” was established in almost every canton. This handwriting style has changed teaching methods, because the children now start with a beginners’ alphabet of printed letters, then learn how to connect the most common letters fluently by garlands (joins on the baseline). Finally, students get support to develop their individual handwriting style (Jurt Betschart & Hurschler Lichtsteiner, 2017). In the German part of Switzerland, no studies about handwriting instruction nor any implications on language acquisition and text quality in particular have been published so far.

### Hypotheses

1. With respect to the different trainings carried out over 5 weeks, we expected that the combined handwriting/spelling training would improve the fluency of handwriting (speed and automaticity) and advance correct spelling as well as the text quality (i.e. number of ideas, text organization) to a higher degree than the handwriting only or a spelling only training because of the broader approach of the combined training, which included two transcriptions skills.
2. In addition, we expected that the handwriting only and the spelling only training groups would both outperform the reading control group regarding handwriting fluency, spelling and text quality. We also expected that the aforementioned group differences would sustain over a period of 3 months after termination of the intervention.
3. Irrespective of the trainings, we expected that text quality would be independently affected by handwriting fluency and automaticity, spelling, working memory, and gender. Text quality is assumed to be higher among children, indicating higher handwriting fluency and automaticity, improved spelling and working memory capabilities. In addition, text quality is assumed to be higher among girls than among boys.

## Method

### Design

**Fig. 1:**
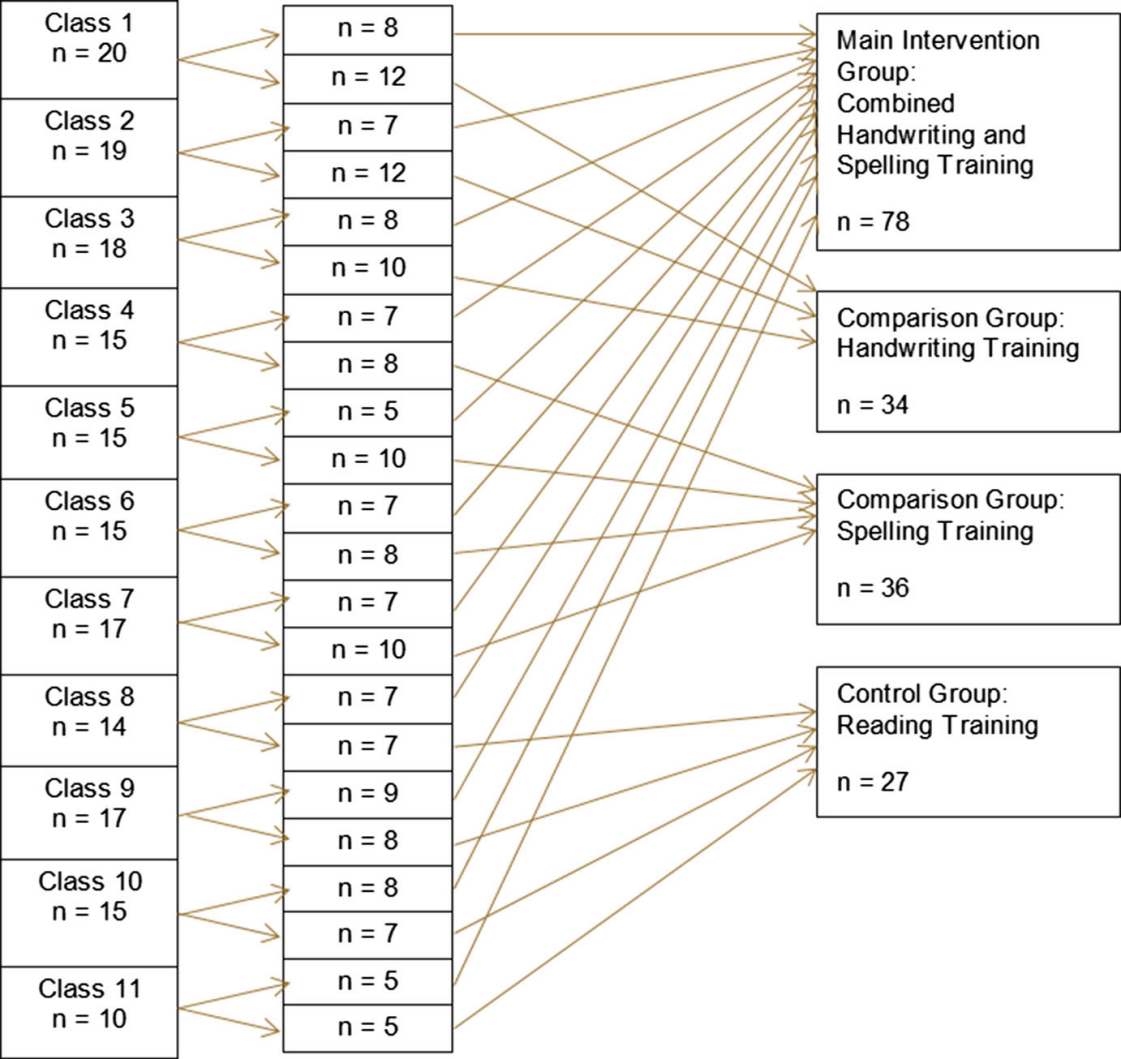
Assignment of children to the groups, N = 175

A randomized controlled field study with pre-test, post-test and follow-up-test measurements was conducted. The sample included one main intervention group (a combined handwriting and spelling training), two comparison groups (handwriting training and spelling training) and a control group (reading fluency). The participants were typically developing third-graders (N = 175). This sample size was higher than the required sample size (N = 158) calculated by means of the GPower software (Faul, Erdfelder, Lang, & Buchner, 2007) assuming an effect size f(V) = 0.26 and alpha error = 0.05 (given 4 groups and 3 measurements, and repeated measures ANOVA). In order to control for cluster effects, each class was randomly divided in two parts, whereby the minority of every class was allocated to the main intervention and the majority randomly to one of the comparison groups or to the control group (Fig. 1). Most measures were assessed three times, however, working memory capacity and visual-motor integration were assessed only during the pre-test.

### Participants

Principals from elementary schools in the Canton of Lucerne in the German speaking part of Switzerland were asked to help the research team in recruiting the participating classes. The participating teachers had to give their consent of attending to four preparatory meetings and organizing the testing and training activities. They also had to find a second teacher for the training phase who was usually the associated teacher for special needs education.

All children in the classes were included in the curriculum-based trainings. 202 children and their parents were asked to give their active consent for the additional tests and the use of the data; in 21 cases, the consent was refused. Because the average number of pupils in each class was lower than expected, it took 11 instead of 9 classes to reach the necessary sample size. 6 of the 181 participants moved away during the intervention time, which resulted in 175 children who attended the whole training program and all of the tests (attrition = 3.3%). Within the participants, there were no exclusions due to learning disabilities or other issues, but in order to control for special effects, all possible variables were noticed. 22 children (12.6%) attended integrative special needs education; 11 children (6.3%) received therapies such as psychomotor therapy. These cases were evenly distributed over the four intervention groups. 20 children (11.4%) attended L2 courses in German with uneven distribution among the groups: 17 of them were in the combined handwriting and spelling or in the handwriting group; due to the low numbers, a statistical test of this difference was not possible. Regarding age *(M*_age_ = 9.0 years, *SD* = 0.4 years, age range = 7.5–10.2 years), the children of the four intervention groups did not differ significantly. Demographic characteristics such as gender, handedness and language spoken were equally distributed over the four groups (see Table 1).

**Table 1:** Demographic characteristics of the sample

Variable			Combined		Handwriting		Spelling		Reading			
	N	%	n	%	n	%	n	%	n	%	χ^2^	p
	175		78		34		36		27			
Gender											0.599	n.s.
Female	89	50.9	38	48.7	18	52.9	20	55.6	13	48.1		
Male	86	49.1	40	51.3	16	47.1	16	44.4	14	51.9		
Handedness											6.076	n.s
Right handers	157	89.7	74	94.9	28	82.4	30	83.3	25	92.5		
Left handers	18	10.3	4	5.1	6	17.6	6	16.7	2	7.4		
Languages											4.879	n.s.
Swiss German speaking only	129	73.7	61	78.2	21	61.8	29	80.6	18	66.7		
Two or more languages	46	26.3	17	21.8	13	38.2	7	19.4	9	33.3		

### Materials (digitizer)

Handwriting measures were recorded and analyzed by means of the software package CSWin (Mai & Marquardt, 2007) on a personal computer (Lenovo ThinkPad T61p Type 6457-7WG), which was connected to a digitizing tablet (Wacom Intuos 4 Pen Tablet PTK-640 and Intuos Inking Pen). The sampling frequency was 200 Hz and the accuracy was 0.1 mm in both x and y directions. Position data were recorded even if the stylus was lifted (less than 10.0 mm) above the tablet due to the inductive method of measurement. Nonparametric regression methods (kernel estimation) are part of the mathematical procedures of the program (Marquardt & Mai, 1994) in order to calculate and to smooth velocity and acceleration signals.

### Intervention

7 months prior to intervention, four meetings with the participating teachers took place: All participants had to plan the same quantity and quality of instruction and training regarding writing, German orthography and handwriting training throughout the curriculum. In addition, every teacher got an introduction into the didactic structure of the handwriting lessons as well as the materials necessary to prepare standardized lessons in the time period from summer 2015 to the end of January 2016 when the intervention started.

The participating teachers (n = 11) and their students were randomly assigned to the different intervention and control conditions, which lasted 20 units (15 min per unit) distributed over 5 weeks (4 units/week). The respective trainings were highly standardized and held during ordinary school hours by the participating teachers who had been instructed in advance. The teachers had to check for an implementation as full as possible. Within the materials, the last page (for the last day) included a feedback sheet for the participating pupils in order to assess their motivation.

We know that effective handwriting training should always be followed by meaningful tasks like writing or at least exercises including language actions (playing with words, little learning games like riddles) (Alves et al., 2015; Berninger & Richards, 2002). However, trainings for handwriting skills often include (sometimes unintended) orthographic challenges and spelling training can be very demanding for some children when it takes a lot of handwriting. This is why we tried to separate these training conditions from the beginning. Even the combined training offered a simple start without cognitive overload followed by different transfer opportunities.

#### Combined handwriting and spelling training

Completely new training materials were developed for the combined intervention and for the handwriting intervention as well. Due to the semi-cursive handwriting type used in the German-speaking part of Switzerland, the material did not focus on the repetition of the alphabet (since the production of individual letters is presumed to be already automatized at that age), but on the fluent production of the connections between the letters, specifically on the connections as realized by garlands (joins on the baseline). According to Berninger and Richards (2002), the training was planned to be taught explicitly in a regular manner, by providing short and daily trainings of 15 min followed by writing activities. The daily intervention program was presented by a two-sided sheet with two A4-sized pages of material on each side starting with a finger-warm-up exercise (scribbling strokes and circles) followed by a graphomotor training of a selected pair of letters to be joined fluently under different conditions regarding speed, pressure, size and visual control according to Frank, Michelbrink, Beckmann and Schöllhorn (2008). Every week, a group of similar patterns (e.g. day 1 e-n, day 2 e-m, day 3 e-r) was offered. The last day was dedicated to repetitions of this group. These trainings had to be completed in broad colorful bands instead of rulings. In the combined handwriting and spelling condition, the task in the second part was to put these patterns of 2–3 joined letters into use within a spelling task, which was a training mostly focusing on morphemes, or to use these patterns directly by writing words or sentences. To make sure that the children spent enough time on the language-based part the last 5 min had to be strictly spent on the pages of the second part.

#### Handwriting only training group

In the handwriting group, the tasks consisted of a graphomotor training (the same as page 1 and 2 of the combined group supplied with additional tasks of that type) without any combination with spelling training.

#### Spelling only training group

The spelling training was based on a training developed by Leemann (2015). We applied the chapter focusing on morphemic structures (Leemann, 2015, pp. 19–44), which had been described as the third of three important principles of German orthography (Eisenberg & Feilke, 2001) and is part of the regular curriculum at that age. Exercises consisted of recognizing morphemic elements, analyzing words and constructing them by using morphemes. In order to avoid handwriting within these tasks, the children had to complete them without handwriting, e.g. by marking morphemes using text markers or connecting questions and solutions by cues.

#### Reading training control group

The training for reading fluency consisted of two parts (reading skills and reading fluency training) of the training developed by Kruse, Rickli, Riss, and Sommer (2014). Handwriting activities were omitted during these trainings.

The intervention protocols indicated a mean number of training units of 19.3 (SD = 1.4) for the total sample. The mean number of training units varied from 19.0 to 19.8 over the four groups and did not differ significantly from each other.

### Procedure

Data collection of the pre-test took place at the end of January 2016. The intervention started midst February and lasted 5 weeks. During the subsequent week, the post-tests were carried out. After a break of 11 weeks, the data collection of the follow-up-test took place midst June 2016. Most tests were group-administered by the teachers (spelling test, copy task, writing a 30’-text, visual motor integration), whereas two tests (Working Memory Test AGTB-5, Hasselhorn et al., 2012; and CSWin, Mai & Marquardt, 2007) were executed as single tests by the data collection team consisting of two researchers and five scientific assistants. Legibility of the handwriting was assessed by analyzing the copied texts by a rating system; however, since this variable is not focused on in this paper, we do not describe this measure in detail in this study.

### Tasks and measures

#### Personal details of participants

Details on children’s handedness, gender, age, languages spoken were collected by the participating teachers and submitted to the research team.

#### Handwriting fluency measured on the digitizer

The handwriting tasks were administered on a digitizing tablet and included a range of 17 items starting with basic handwriting movements, letter, and combinations of two joined letters, syllables, morphemes, non-sense words and common words. The children also had to copy sentences, carry out a small dictation and write two separate 3-min short texts. The first was about a fantasy theme (“If I could conjure”, “If I were rich”, “If I were a super star”), the second was a prescription (“This is my way to school”, “My favorite game”, “My favorite pet”). All tablet items were written with a regular inking pen on a blank sheet of paper placed on the digitizing tablet.

Within each task, we derived the number of inversions in velocity (NIV) and the stroke frequency (FREQ) from the digitizer. The number of inversions in velocity (NIV) indicates the average number of velocity changes occurring within writing strokes. Fluent handwriting requires only one velocity change per stroke (acceleration followed by deceleration) and therefore results in a NIV score that is approximately or exactly equal to 1, whereby 1 indicates perfect automaticity (Mai & Marquardt, 1999). When copying a sentence, the mean number of inversions in velocity of 4th graders amounted to 2.7 (SD = 2.1) in an earlier study (Hurschler, Saxer, & Wicki, 2010). Frequency (FREQ) refers to the number of upward and downward movements in 1 s calculated by CSWin (Mai & Marquardt, 1999). This measure seems more appropriate than assessing speed as stroke length per second (mm/s) (the latter strongly depends on a person’s writing size). In our earlier study, the mean stroke frequency of 4th graders was f = 2.1 (SD = .6) when copying a sentence (Hurschler et al., 2010).

#### Handwriting measures irrespective of tablet tasks

The children had to execute a 5 min copying task where they were asked to write as fast and as legible as possible. Speed of handwriting was measured as the number of letters written within that task. The respective data of one class had to be excluded from the analysis because of violating the time limit.

#### Visual motor integration VMI (Beery & Beery, 2006)

The Developmental Test of Visual-Motor Integration (VMI) “is designed to assess the extent to which individuals can integrate their visual and motor abilities” (Beery & Beery, 2006, p.14). It consists of 30 graphic forms of increasing complexity that are to be copied successively. The scoring procedure follows the instructions as specified in the test manual.

#### Working memory

At pre-test (t1), we administered three working memory sub-tests from the AGTB 5-12 (Hasselhorn et al., 2012). In addition to the two visual-spatial tasks (Corsi-Block and Matrix), a third task referring to the phonological loop (word-list recall-test, monosyllabic words) was administered.

#### Spelling

In order to get three parallel test versions for spelling, we combined the age-appropriate items of the “Salzburger Rechtschreibetest: SLRT-II” (Moll & Landerl, 2014) with some age-appropriate items from the “Diagnostischer Rechtschreibtest DRT 3” (Müller, 2003). The resulting test versions were pilot tested in advance revealing comparable test difficulties.

#### Text quality

The children were asked to write short experimental narrative texts according to a prompt (adapted from Kim et al. 2015). The three prompts linked to the pre-, post- and follow-up test were familiar to their everyday life (“This happened when I got home from school…”, “This happened in our break…”, “This happened when I was a child…”) and were presented in a randomized order for each class. Children were allowed to write a brief mind-map of their ideas before writing. They were told that this time, their drafts would be the piece that would be evaluated and that there would be no phases of revision and/or clean copy. Each time, the teacher told the children the principal criteria of the later assessment in advance. The four criteria (1. My story has a gripping beginning, a middle part and a clear ending; 2. It is coherent without disruptive leaps; 3. It is told in a vivid and exciting way; 4. I use appropriate matching words and sentences) were derived from the curricular teaching materials. There was no help allowed by the teacher or by peers during production. After 30 min, all products—drafts and pieces of planning work—were collected and sent to the research team.

The text quality was assessed by a rating procedure based on two German studies, Ko-Text (Kruse, Reichardt, Herrmann, Heinzel, & Lipowsky, 2012) and RESTLESS (Wild et al., 2016), meeting our requirements with respect to grade level and text type (narrative texts). The original rating consisted of 10 Likert-type items complemented by anchor examples. After the instruction of 6 raters and preliminary interrater tests, the applied rating system consisted of 7 items (coherence of topic, logic of action, cohesion, implicitness of the text, appropriate language, orientation towards basic patterns of narrative texts, and language risk). These items are outlined in detail in the Appendix. The text quality measure was calculated by two steps. First we combined the three coherence items (coherence of topic, logic of action, cohesion) into one coherence measure. Subsequently we averaged coherence and the remaining four items. In order to assess the interrater reliability, 19 texts (of the pre-test) were rated by all raters. Based on the average ratings, we calculated the intraclass correlation (ICC) average score by applying the two-way model. The resulting ICC of 0.97 indicates a good reliability.

#### Motivation and self-evaluation

Integrated in the last training sheet, the children were asked to complete a short survey of self-evaluation relating to level of program difficulty and their motivation to participate and were given the possibility to write an open formatted feedback.

### Data analysis

The hypotheses with respect to the intervention effects were examined as group × time interactions within the longitudinal data set (repeated measurements). We calculated them by means of repeated measures ANOVAs. Some departures from sphericity were identified by the Mauchly’s test in SPSS. Since all violations were weak (epsilon > 0.75), we applied the Huynh–Feldt correction.

As the handwriting measures derived from the digitizer (number of inversions in velocity and stroke frequency) were skewed and not normally distributed (as confirmed by Shapiro–Wilk tests), we calculated the logarithmic (base 10) values of these measures to meet the requirements for parametric statistics outlined before.

## Results

In this section, we will first describe the level of fluency and automaticity of handwriting being revealed in the different tasks used in this study. Secondly, we will report the results concerning the intervention effects, and thirdly we will present our results with respect to the hypotheses that are irrespective of the intervention.

### Fluency of handwriting among third graders

As the original values (before logarithmic transformation) are easier to understand (what these figures really mean), we will depict the means and standard deviations of the untransformed values in Table 2.

**Table 2:** Fluency of handwriting across time

Measure	t1		t2		t3	
	M	(SD)	M	(SD)	M	(SD)
NIV 1	1.54	(1.00)	1.24	(0.47)	1.23	(0.57)
FREQ 1	3.34	(1.17)	3.69	(0.95)	4.02	(1.00)
NIV 2	2.74	(3.17)	2.04	(2.21)	1.59	(0.88)
FREQ 2	1.80	(0.74)	2.06	(0.75)	2.26	(0.85)
NIV 3	1.52	(1.28)	1.49	(2.98)	1.10	(0.35)
FREQ 3	3.01	(1.14)	3.40	(1.00)	3.62	(0.91)
NIV 4	1.26	(0.55)	1.35	(0.69)	1.35	(0.69)
FREQ 4	2.91	(1.06)	2.71	(1.07)	2.61	(1.05)
NIV 5	1.13	(0.37)	1.13	(0.40)	1.07	(0.17)
FREQ 5	3.71	(1.20)	3.71	(1.20)	3.63	(1.03)
NIV 6	1.25	(0.65)	1.18	(0.44)	1.23	(0.51)
FREQ 6	2.99	(0.76)	3.08	(0.72)	3.05	(0.77)
NIV 7	1.09	(0.18)	1.06	(0.12)	1.04	(0.09)
FREQ 7	3.63	(0.71)	3.85	(0.70)	3.93	(0.62)
NIV 8	2.41	(0.18)	2.32	(1.46)	2.12	(1.31)
FREQ 8	1.88	(0.62)	1.82	(0.55)	2.02	(0.57)
NIV 9	3.25	(2.25)	2.17	(1.27)	2.37	(1.48)
FREQ 9	1.54	(0.53)	1.81	(0.51)	1.86	(0.56)
NIV 10	2.56	(1.48)	2.05	(1.26)	1.83	(0.92)
FREQ 10	1.82	(0.57)	1.99	(0.58)	2.22	(0.66)
NIV 11	2.23	(1.27)	2.10	(1.11)	1.83	(1.00)
FREQ 11	2.08	(0.64)	2.12	(0.67)	2.46	(0.75)
NIV 12	1.78	(1.02)	1.73	(1.03)	1.55	(0.82)
FREQ 12	2.51	(0.80)	2.45	(0.82)	2.80	(0.88)
NIV 13	1.69	(0.71)	1.48	(0.53)	1.37	(0.45)
FREQ 13	2.49	(0.62)	2.65	(0.59)	2.91	(0.58)
NIV 14	1.58	(0.64)	1.48	(0.51)	1.38	(0.37)
FREQ 14	2.65	(0.68)	2.70	(0.60)	3.01	(0.58)
NIV 15	1.63	(0.66)	1.52	(0.58)	1.38	(0.43)
FREQ 15	2.62	(0.66)	2.70	(0.67)	3.03	(0.61)
NIV 16	1.24	(0.25)	1.22	(0.25)	1.18	(0.16)
FREQ 16	3.52	(0.67)	3.59	(0.67)	3.91	(0.62)
NIV 17	1.44	(0.56)	1.40	(0.41)	1.29	(0.30)
FREQ 17	2.92	(0.64)	2.94	(0.63)	3.24	(0.56)

N = 174 − 175

NIV = Number of Inversions in Velocity; FREQ = Stroke Frequency; 1 = scribbling; 2 = finger movements; 3 = finger movements-faster; 4 = wrist movements; 5 = wrist movements (faster); 6 = combined finger and wrist movements; 7 = combined finger and writ movements (faster); 8 = garlands; 9 = double loops; 10 = syllables; 11 = nonsense words; 12 meaningful words; 13 = 3′ composition (based on a preliminary version); 14 = 3′ composition (descriptive text-without a draft); 15 = sentence writing (normal pace); 16 = sentence writing (as fast as possible); 17 = sentence dictation

According to Marquardt (2011), a handwriting movement is almost automated if the number of inversions in velocity is lower than 1.5. Regarding the data at the pre-test, it is remarkable that for some tasks, the children did already show automatized movements. As expected, they very well managed to perform the simplest basic movements as wrist movements (NIV 4, NIV 5) and combined finger and wrist movements (NIV 6, NIV 7). This is not the case in regard to patterns like garlands (NIV 8) and double loops (NIV 9) or while writing syllables (NIV 10), words (NIV 11, NIV 12) and composing short texts (NIV 13, NIV 14). However, when asked to write a sentence as fast as possible (NIV 16) or to write a difficult sentence by dictation (NIV 17), they achieved very low values indicating high automaticity.

Over time, almost all movements were at an automatized level at t2, even the short texts, except at the first trial of finger movements (NIV 2) and the garlands, double loops, syllables and words. In general, a clear linear improvement over time is discernible by visual inspection only.

The stroke frequencies mirror the number of inversions in velocity findings described above: They are in general already high from the beginning of the study at pre-test and further improve from t1 to t3.

### Intervention effects

In order to avoid a multiple testing problem (accumulation of alpha error), we restricted the testing of the expected intervention effects on fluency to the movements (tasks) that were not yet fully automated at t1, i.e. double loops, a syllable (i.e. “neu”), words (i.e. “akir” and “Falle”) and the two composed texts. Thus, twelve repeated measures ANOVAs were separately carried out using the fluency measures (automaticity and frequency) of six of the mentioned tasks as dependent variables. We found reliable time effects in almost all analyses but-with two exceptions—no intervention effects. The three intervention groups, as well as the control group, wrote more fluently (i.e. with improved automaticity, higher stroke frequency) over time. For example, the automaticity of handwriting (measured by the NIV score) when composing a text improved significantly over time (*F*(1.8,306.9) = 26.9, *p* < 0.001, η_p_^2^ = 0.14), however, in general, there was no group*time interaction, i.e. the improvements of the combined intervention group did neither outperform the improvements of the spelling only nor the handwriting only training group nor did they differ from the improvements of the reading control group.

**Fig. 2:**
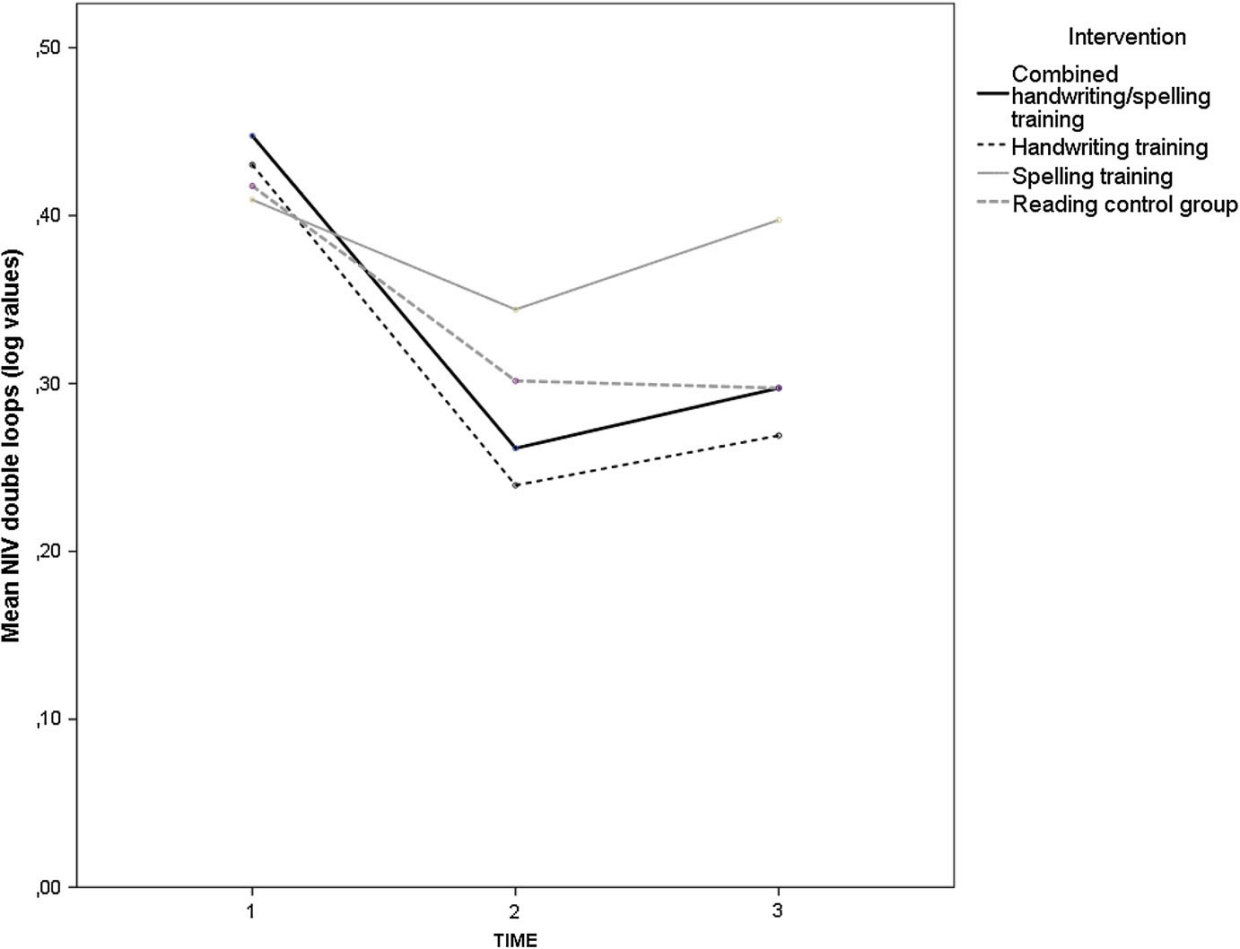
Course of automaticity (double loops) among interventions groups

One of the exceptions mentioned above was a marked increase of fluency in the double loops tasks observed in the combined and in the pure handwriting group from pre-test to post-test, which outperformed the spelling training and the reading groups as indicated by the significant interaction term intervention*time (*F*(3,171) = 2.7, *p* < 0.05, η_p_^2^ = 0.05). As depicted in Fig. 2, the differences did not persist completely until the follow up, resulting in a nonsignificant interaction term when the third measurement was also included in the model.

The second exception concerned stroke frequency when completing the task referring to writing the German word “Falle”. Here, we found an intervention × time interaction (*F*(6,342) = 3.8, *p* = 0.001, η_p_^2^ = 0.06) besides an intervention effect (*F*(3,171) = 3.2, *p* < 0.05, η_p_^2^ = 0.05) and a time effect (*F*(2,342) = 12.3, *p* < 0.001, η_p_^2^ = 0.07). Interestingly enough but completely unexpected, the respective means indicated that the stroke frequency declined among the combined and the handwriting training group from t1 to t2 and recovered from t2 to t3, i.e. showing a paradox intervention effect, while the spelling group and the reading group did improve their stroke frequency quite linearly over time.

With respect to our hypothesis that assumed intervention effects on spelling and text quality, we calculated two additional repeated measures ANOVAs including this time spelling and text quality respectively as dependent measures. Again, these analyses failed to discern any group differences or intervention effects. However, we found a time effect showing that text quality had improved over time (*F*(2,338) = 6.1, *p* < 0.01, η_p_^2^ = 0.04).

### Predicting text quality irrespective of the intervention

Our third hypothesis relates to the prediction of test quality regardless of group membership. We calculated a hierarchic regression analysis on text quality (measured at t3) by entering gender as the first predictor. In the second step, we entered two working memory measures (t1) and visuo-motor integration (t1) and in the final step, we entered the transcription measures, i.e. number of inversions in velocity (t3) and speed (t3) as well as spelling (t3). In order to investigate the influence of a clear-cut low automaticity, the number of inversions in velocity measure used for that analysis was dichotomized above one standard deviation (see Table 1). The final regression equation was significant indicating that the included variables are able to predict text quality (adj r^2^ = 0.21). The results presented in Table 3 reveals that gender, the phonological part of the working memory, spelling and the dichotomized NIV contributed to this prediction while the visual part of the working memory, visuo-motor integration and writing speed did not.

**Table 3:** Summary of hierarchical regression analysis for variables predicting text quality

Variable	Model 1			Model 2			Model 3		
	*B*	*SE B*	*β*	*B*	*SE B*	*β*	*B*	*SE B*	*β*
Gender	− 0.27	0.07	− 0.28**	− 0.29	0.07	− 0.31**	− 0.23	0.07	− 0.25**
WM word span				0.20	0.05	0.26**	0.15	0.05	0.20**
WM visuo-spatial sketch pad				− 0.00	0.02	0.02	0.00	0.03	0.02
VMI				0.02	0.01	0.08	0.02	0.01	0.09
Handwriting speed							0.00	0.00	0.02
NIV Composing							− 0.35	0.15	− 0.17*
Spelling							− 0.02	0.00	− 0.23**
*R* ^*2*^	0.08			0.16			0.20		
*F* for change in *R*^*2*^	14.90**			5.35**			6.03**		

NIV composing is a dichotomized variable

*WM* working memory; *VMI* Visuo-motor integration

*p < 0.05. **p < 0.01

## Discussion

### Intervention effects

Third graders’ handwriting performance is interesting to analyze as they are in the midst of the learning process: They are more advanced than real beginners because they have mastered the first step of using the corresponding link from phonemes to graphemes and they know how to form the letters, but are not yet fully automatized since they are not able to write words and sentences without paying attention as skilled writers do. Therefore, this intervention study was designed to investigate the impact of a training program that aims to improve the transcription skills of third graders. Improved fluency is assumed to have a positive impact on text quality as previous studies have demonstrated.

In line with our assumptions, we could only demonstrate a short-term intervention effect on fluency with respect to the automaticity improvements of double loops that, however, did not persist until t3. Besides that, the intervention did not improve fluency among the two handwriting groups to greater extent than among the remaining groups (spelling and reading). In contrast, we found a paradox effect indicating that the handwriting training could also have contributed to a reduction of fluency as evidenced by the results related to the task of writing a meaningful word. It is likely that the handwriting training temporarily promoted more deliberate movements among some children and not more automaticity in the first place.

Another explanation for the missing intervention effect is the fact that the children’s automaticity e.g. when writing sentences was already very well developed at the beginning of the study. Irrespective of whether they really realized the promoted garlands as a principle to increase handwriting fluency in their own handwriting (which would be an interesting qualitative further investigation), after reaching a certain automaticity in their own personal style, to learn and integrate new patterns could interfere with further improvements of fluency in the short-term. Under this premise, further improvements could not so easily be achieved by means of a training lasting 5 weeks or 20 units. Furthermore, it is quite difficult to prevent a control group like our reading group from handwriting “training” over 5 weeks of schooling since handwriting is part of everyday activities beyond the intervention units. However, it is possible that a program lasting longer than 5 weeks and more training units can enhance transcription skills among third graders as was the case in the study conducted by Alves et al. (2015).

As the intervention did not yield differential effects on fluency, it is theoretically congruent that we also did not find any intervention effects on text quality since the former was thought to be a precondition of the latter.

The fact that we did not find an effect on the spelling training is perhaps less surprising. The improvements in spelling develop slowly and are hardly measurable within several weeks.

### Automaticity of handwriting, working memory, spelling, and text quality

As outlined above transcription skills facilitate composing especially among young children because at that age handwriting is not yet automated and therefore working memory resource consuming (Graham, Berninger, Abbott, Abbott, & Whitaker, 1997). In line with this assumption the multiple regression analysis revealed that text quality positively was both related to the phonological loop component of working memory and to transcription measures such as fluency and spelling. In some contradiction with previous research, however, text quality was independent from the visuo-spatial component of working memory. With respect to transcription, interestingly, speed writing per se did not predict text quality whereas it was a dichotomized variable consisting of very low automaticity versus normal to good automaticity (measured as number of inversions of velocity) when writing a short text that emerged as an independent predictor of text quality.

Thus, children whose cognitive resources are consumed by the transcription process because of very low automaticity and reduced spelling capacity lack that capacity when composing. This, in turn, is one possible reason for low text quality, a finding that is consistent with Prunty, Barnett, Wilmut and Plumb (2016), who investigated children with developmental coordination disorder, although they used other measures of handwriting fluency.

### Gender

Gender successfully predicted text quality. Referring to gender it is well known that boys’ reading fluency competencies are lower than girls’ (Bos, Tarelli, Bremerich-Vos, & Schwippert, 2012), and also the text quality of boys seems to be constantly lower than that of girls (Kim et al., 2015). Thus, we replicated previous results and controlled for the gender effect as we entered gender first into the regression.

## Conclusion

According to Santangelo and Graham (2016), teaching handwriting is an important duty over all primary school years. Although they advocated an individualized instruction taking into account different stages of graphomotor development and the capabilities to deal with new patterns, our design followed an experimental logic by controlling as many factors as possible. Therefore, we have to admit that our trainings among third graders do not fulfil the demands of an individualized instruction.

As a recommendation to the involved schools and to teachers of third graders, we therefore propose to start the teaching on how to join letters by garlands earlier (that means at the beginning of the third class) when children are not yet fully automatized in their handwriting, to observe learning progress carefully and to offer suitable individualized training as it is realized in the official materials in the meanwhile (Jurt Betschart, Hurschler Lichtsteiner, & Reber, 2016).

## Appendix: items of the instrument measuring text quality

Each item has 4 levels. The lowest and the highest levels are detailed below. The full rating system is made available by the first author.

**Table:**

Item	Coherence of topic (1st dimension measuring coherence)
Level 1	It is difficult to determine the topic of the text.
Level 4	A dominant topic of the text can be determined directly without any problems.
	If there are two topics, both topics must be formulated clearly and comprehensively.

Item	Logic of action (2nd dimension measuring coherence)
Level 1	No recurring theme can be recognized in the text. The episodes of actions are structured in a way that do not allow for recognizing any logic of action. Text parts are very loosely related to each other.
Level 4	A recurring theme can be recognized very clearly and easily. Episodes of actions are structured in a way that allows for recognizing logic of action throughout the text. Text parts build on one another in the correct order and are very well linked to each other.

Item	Cohesion (3rd dimension measuring coherence)
Level 1	Temporal relations are not supported by linguistic devices or linguistic devices are used incorrectly.
Level 4	Temporal relations are supported by at least three correctly used linguistic devices, whereby two of them must be different. “And” as a temporal device must not be one of them. (It may occur as an additive device, though).

Item	Implicitness of the text
Level 1	Throughout the discourse it is often *not uttered* what should be said in order to enable the reader to follow the events. Intermediate steps need to be introduced by the reader to complete the meaning of the text or it is barely possible to complete the text meaningfully. Even though agents can be recognized within the text, their actions relared to one another are not plausible.
Level 4	*Everything* is uttered what needs to be said in order to let the reader follow the events unfold *without any problems*. The text can be *fully* understood. No completions are necessary. Agents can be recognized easily and their actions related to each other appear to be very plausible.

Item	Appropriate language
Level 1	A great many mistakes occur when it comes to word formation, syntax and the use of function words. The incorrect use of linguistic devices often impedes the construction of meaning.
Level 4	Hardly any mistakes occur when it comes to word formation, syntax and the use of function words. The incorrect use of linguistic devices does not impede the construction of meaning. Words and sentences are used appropriately.

Item	Orientation towards basic patterns of narrative texts
Level 1	No storyline can be recognized. No basic narrative patterns (change of plan, complication, tension, punch line) can be recognized.
Level 4	A complete storyline can be recognized. All parts of basic narrative patterns (change of plan, complication, tension, punch line) can be recognized, and they are equally well developed.

Item	Language risk
Level 1	No formal linguistic risk is taken.
Level 4	A formal linguistic risk is taken in four or more cases.
